# “Orphan” Connexin43 in Plakophilin-2 Deficient Hearts Revealed by Volume Electron Microscopy

**DOI:** 10.3389/fcell.2022.843687

**Published:** 2022-03-08

**Authors:** Chantal J. M. van Opbergen, Joseph Sall, Chris Petzold, Kristen Dancel-Manning, Mario Delmar, Feng-Xia Liang

**Affiliations:** ^1^ The Leon H. Charney Division of Cardiology, New York University Grossman School of Medicine, New York, NY, United States; ^2^ Microscopy Laboratory, Division of Advanced Research Technologies, New York University Grossman School of Medicine, New York, NY, United States

**Keywords:** arrhythmogenic right ventricular cardiomyopathy, plakophilin-2, serial block-face scanning electron microscopy, volume electron microscopy, connexin43 hemichannels, nanogold

## Abstract

Previous studies revealed an abundance of functional Connexin43 (Cx43) hemichannels consequent to loss of plakophilin-2 (PKP2) expression in adult murine hearts. The increased Cx43-mediated membrane permeability is likely responsible for excess entry of calcium into the cells, leading to an arrhythmogenic/cardiomyopathic phenotype. The latter has translational implications to the molecular mechanisms of inheritable arrhythmogenic right ventricular cardiomyopathy (ARVC). Despite functional evidence, visualization of these “orphan” (i.e., non-paired in a gap junction configuration) Cx43 hemichannels remains lacking. Immuno-electron microscopy (IEM) remains an extremely powerful tool to localize, with nanometric resolution, a protein within its native structural landscape. Yet, challenges for IEM are to preserve the antigenicity of the molecular target and to provide access for antibodies to reach their target, while maintaining the cellular/tissue ultrastructure. Fixation is important for maintaining cell structure, but strong fixation and vigorous dehydration (as it is routine for EM) can alter protein structure, thus impairing antigen-antibody binding. Here, we implemented a method to combine pre-embedding immunolabeling (pre-embedding) with serial block-face scanning electron microscopy (SBF-SEM). We utilized a murine model of cardiomyocyte-specific, Tamoxifen (TAM) activated knockout of PKP2. Adult hearts were harvested 14 days post-TAM, at this time hearts present a phenotype of concealed ARVC (i.e., an arrhythmogenic phenotype but no overt structural disease). Thick (200 µm) vibratome slices were immunolabelled for Cx43 and treated with nanogold or FluoroNanogold, coupled with a silver enhancement. Left or right ventricular free walls were dissected and three-dimensional (3D) localization of Cx43 in cardiac muscle was performed using SBF-SEM. Reconstructed images allowed us to visualize the entire length of gap junction plaques, seen as two parallel, closely packed strings of Cx43-immunoreactive beads at the intercalated disc. In contrast, in PKP2-deficient hearts we observed bulging of the intercellular space, and entire areas where only one of the two strings could be observed, indicating the presence of orphan Cx43. We conclude that pre-embedding and SBF-SEM allowed visualization of cardiac Cx43 plaques in their native environment, providing for the first time a visual complement of functional data indicating the presence of orphan Cx43 hemichannels resulting from loss of desmosomal integrity in the heart.

## Introduction

Plakophilin-2 (PKP2) is an abundant protein of the desmosome, an intercellular adhesion structure (Grossmann et al., 2004). Mutations in PKP2 associate with most cases of gene-positive arrhythmogenic right ventricular cardiomyopathy (ARVC), a pleiotropic disease that can manifest as mainly electrical, structural or both depending on stage progression (Ohno, 2016). As such, while ARVC is best recognized as a cardiomyopathy of right ventricular predominance, catecholaminergic sudden cardiac arrest is common during the subclinical (or “concealed”) phase of the disease (Cadrin-Tourigny, 2019; Müssigbrodt et al., 2019). The molecular/cellular mechanisms responsible for these arrhythmias remain unclear and current treatment options are still lacking. Recent studies have demonstrated that in addition to cell-cell adhesion, PKP2 and its intercalated disc (ID) partners translate information initiated at the site of cell-cell contact into intracellular signals that maintain structural and electrical homeostasis (Cerrone et al., 2017; Austin et al., 2019).

To study the role of cardiomyocyte PKP2 expression in cardiac function, we developed a cardiomyocyte-specific, tamoxifen (TAM)-activated, PKP2 knockout murine line [PKP2cKO; (Cerrone et al., 2017)]. Adult PKP2cKO mice present, in a compressed timeline, aspects of the history of human ARVC. Following TAM injection these hearts progress from a normal state, to an arrhythmogenic cardiomyopathy of right ventricular (RV) predominance and eventually biventricular dilated cardiomyopathy and end-stage failure (Cerrone et al., 2017). In Kim et al., PKP2cKO mice were studied 14 days post-TAM, a time point at which hearts present a phenotype of concealed ARVC (i.e., an arrhythmogenic phenotype but no overt structural disease) (Kim et al., 2019). At this stage, ventricular myocytes present increased Connexin43 (Cx43)-mediated membrane permeability. The latter is considered responsible for an excess entry of calcium (Ca^2+^) into the cells, eventually leading to the arrhythmogenic/cardiomyopathic phenotype.

Gap junction plaque formation is known to depend on proper intercellular adhesion (Beardslee et al., 2000). A recent study from our group showed that PKP2 deficiency can increase cell membrane permeability to ATP, an event prevented by silencing Cx43 expression (Cerrone et al., 2018). In addition, it is known that Cx43 hemichannels (Cx43-Hs) reside in the perimeter of the gap junction plaques (the perinexus) (Rhett et al., 2011) and are capable of flickering with very low probability (Contreras et al., 2003; Verma et al., 2009). We postulate that loss of PKP2 disrupts gap junction plaque integrity, thereby increasing the population of “orphan” Cx43-Hs in the perinexus. The latter would have important translational implications to the molecular mechanisms of inheritable ARVC. Despite functional evidence, visualization of these “orphan” (i.e., non-paired in a gap junction configuration) Cx43-Hs remains lacking.

With the development of confocal microscopy, live cell imaging and super resolution microscopy, the spatiotemporal characteristics of protein landscapes can now be unraveled in high detail. However, fluorescent microscopy has a restricted imaging range within 1 µm of the coverslip, a maximal resolution of ∼10–30 nm and is constrained to a small number of photo switchable fluorophores. Therefore, it lacks the ability to detect subcellular protein structures at sub-diffraction limit resolution. In that regard, is super resolution microscopy not the most optimal tool for detailed reconstruction of for example individual gap junction plaques, of which the intercellular space is between 2–4 nm. Immuno-electron microscopy (IEM) is an extremely powerful tool to localize, with nanometric resolution, a protein within its native structural landscape. Yet, challenges for IEM rely in preservation of antigenicity of molecular targets, accessibility for antibodies, while maintaining the cellular/tissue ultrastructure. Colloidal gold particles as post-embedding immunoprobes, combined with immunoglobulins or immunoglobuline-binding proteins (protein A), have been widely used for subcellular localization of specific molecules using EM (Faulk and Taylor, 1971; Roth et al., 1978). Previous studies show that smaller gold particles provide a higher labeling density, but gold particles with a diameter below 4 nm become hard to distinguish on ultrathin biological specimen under transmission EM (TEM). Therefore, gold particles with a diameter ranging from 5 to 15 nm are most convenient for on section immunolabeling (Slot and Geuze, 1981; van Bergen en Henegouwen et al., 1986; Yokota, 1988). Given the fact that colloidal gold particles have limited penetration capabilities into cells or tissues (even when using smaller size gold particles, such as 1–3 nm in diameter) and lack stability during the labeling process, they are not considered an optimal tool for pre-embedding and volumetric imaging (Hainfeld and Furuya, 1992). Attempts to prepare probes combining fluorescent labeling and colloidal gold for correlative microscopy resulted in limited success over the years, in part caused by restricted tissue penetration capabilities and hampered fluorescence intensity of these probes (De Brabander et al., 1985, De Brabander et al., 1986; Powell et al., 1997).

Nanogold particles provide an effective alternative. Nanogold is a neutral molecule with a diameter of 1.4 nm that is stable in a wide range of pH’s and ionic charges (Hainfeld and Furuya, 1992). Previous studies have shown that these 1.4 nm gold–Fab’ conjugates can penetrate tissue sections up to 40 µm of thickness, providing a perfect approach for pre-embedding immunolabeling (Sun et al., 1995). Fixation is important to maintain cell structure, but strong fixation and vigorous dehydration (as is routine for EM) can alter protein structure, thus impairing antigen-antibody binding. Since immunolabeling is performed on weakly fixed cells or tissues prior to dehydration, nanogold pre-embedding has a maximum antibody-antigen binding efficiency, comparable to light microscopy labeling. By combining fluorescent probes with covalent bound 1.4 nm clusters of gold atoms, FluoroNanogold allows the visualization of any given antigen using correlative microscopy and/or multimodal microscopy (Cheutin et al., 2007; Giepmans, 2008). Therefore, pre-embedding using nanogold or FluoroNanogold is considered one of the optimal tools for volumetric electron microscopy (volume EM, vEM).

In this study our objective was to visualize and examine, in three-dimensions (3D), the gap junction plaque morphology present in the adult murine heart. We used 200 µm thick sections of adult ventricular free walls of control and PKP2cKO cardiac muscle, pre-embedded with nanogold or FluoroNanogold labeling of Cx43. We measured the characteristics of the intercellular space and of the perinexus using a 3D-vEM approach. Our data show that loss of PKP2 expression led to widening of the intercellular space in the gap junction plaque, and increased abundance of unpaired (orphan) Cx43 channels in the gap junction. We conclude that pre-embedding and serial block-face scanning electron microscopy allowed visualization of cardiac Cx43 plaques in their native environment, providing for the first time a visual complement of functional data indicating the presence of orphan Cx43 hemichannels resulting from loss of desmosomal integrity in the heart.

## Materials and Methods

### Animal Model

We utilized a murine model of cardiomyocyte-specific, Tamoxifen activated knockout of PKP2 (PKP2cKO) previously developed in our laboratory (Cerrone et al., 2017). Mice C57BL/6 were treated in accordance to the Guide for Care and Use of Laboratory Animals published by the US National Institutes of Health. Procedures were approved by the NYU IACUC committee under protocol number 160726-03.

### Chemical Fixation, Immunolabeling and Resin Embedding

**FIGURE 1:**
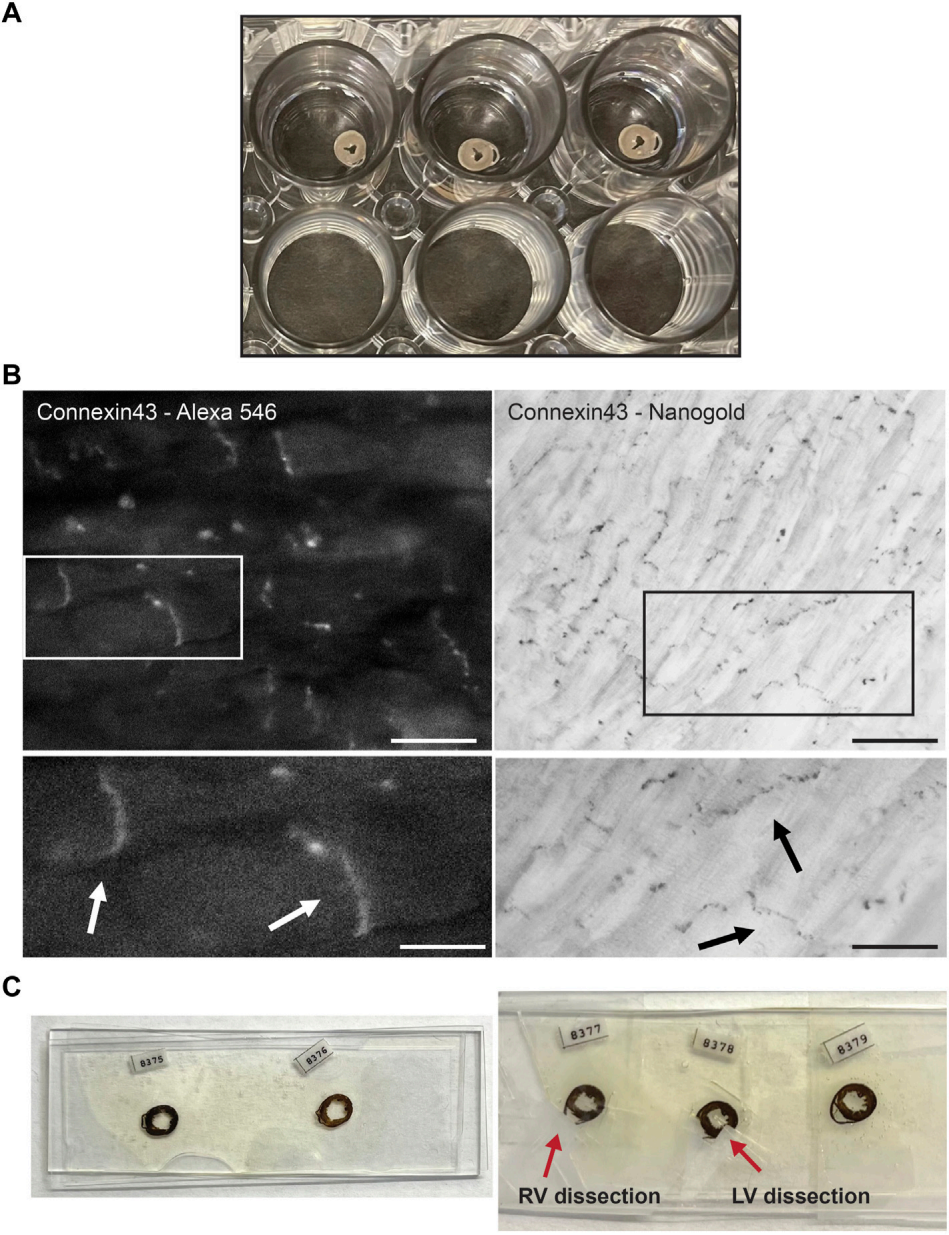
Experimental setup of Connexin43 nanogold labeling in murine cardiac sections. **(A)** Thick cross sections from murine control hearts preserved in 4% paraformaldehyde, stored in a 24 wells plate. Sections are sectioned with a vibratome and of 200 µm thickness. **(B)** FluoroNanogold labeling of adult murine cardiac sections. *Left panel*: FluoroNanogold-546 labeled Connexin43 visualization by light microscopy. Scalebar: 10 µm. Zoom-in at indicated region presented in the panel below, arrowheads depict the intercalated disc (ID). Scalebar: 5 µm. *Right panel*: Nanogold visualization after silver enhancement of Connexin43 by light microscopy. Pictures in both frames are from the same cardiac section. Scalebar: 50 µm. Zoom-in at indicated region presented in the panel below, arrowheads depict the intercalated disc (ID). Scalebar: 10 µm. **(C)** Examples of flat embedded cardiac sections after nanogold labeling. In the left panel the entire cross sections are presented. On the right, two representative examples are shown from dissections of left or right ventricular samples. RV: Right ventricle, LV: Left ventricle.

Control and PKP2cKO mice, 14 days post Tamoxifen injection, were anesthetized by inhalation of 100% CO_2_ and then euthanized by cervical dislocation. After euthanasia, hearts were dissected and perfused through a Langendorf column with a fixative solution of 0.1 M phosphate buffer saline (PBS) and 4% Paraformaldehyde (PFA), then subsequently fixed at 4°C overnight in 4% PFA/PBS. Cross sections of the heart were generated using a vibratome (Leica VT1200S, Buffalo Grove, IL) at 200 µm thickness. Sections were stored in a 24-wells plate (Corning Incorporated, Corning, NY) in 4% PFA/PBS, one section per well (Figure 1A).

For immunolabeling with Cx43, the vibratome sections were washed with PBS and fixed in freshly made 4% PFA and 0.5% glutaraldehyde/PBS for 1 h. After fixation, all steps were performed on a slow speed shaker. The sections were incubated with 50 mM glycine/PBS for 30 min to eliminate unbound aldehydes. Thereafter they were washed in PBS for 15 min and subsequently permeabilized with 0.05% Triton X-100/PBS for 30 min. After blocking with 3% Bovine Serum Albumin (BSA) in PBS for 1 h, the sections were incubated with anti-Connexin43 (Anti-Connexin43 C-terminus cytosolic, affinity purified rabbit polyclonal, Millipore, Cat. #AB1728, 1:50) in 1% BSA/PBS for 2 h at room temperature, after that they were incubated at 4°C overnight. Sections were washed 6 times for 30 min with 1% BSA/PBS, and incubated with Alexa Fluoro^®^ 546 FluoroNanogold™ Fab’ or nanogold Fab’ goat anti-rabbit IgG conjugated secondary antibodies (Nanoprobes, Yanphank, NY, 1:200). Sections were incubated for 2 h at room temperature and then overnight at 4°C. Sections were once more washed with PBS and imaged on an inverted epifluorescence Zeiss AxioObserver microscope with Plan-Neofluar 10x/0.3NA, Plan Apochromat 20x/0.8NA and Plan-Neofluar 40x/0.9NA lenses. For FluoroNanogold labeled tissue sections, fluorescence images were collected with a narrow pass Cy3 filter set, Zeiss part number 43 HE with 550/25 nm exciter, 570 nm dichroic mirror, and 605/70 nm emission filter. After imaging, the sections underwent another fixation step with 2.5% glutaraldehyde/PBS for 2 h, then they were extensively washed with deionized water and 0.02 M sodium citrate buffer (pH 7.0), 3 times for 15 min. Silver enhancement (HQ Silver enhancement kit, Nanoprobes, Yanphank, NY) was performed in the dark for 8 min. Timing of silver enhancement was optimized to maximize the size of gold particles (10–20 nm) without increasing background. Subsequently, sections were washed once with deionized water and imaged with a Zeiss AxiObservor using the brightfield mode. The sections were fixed in 0.5% O_s_O_4_ in aqueous solution for 15 min and stained with 0.5% uranyl acetate in aqueous solution for 1 h in the dark. Sections were dehydrated in a series of ethanol, exchanged with acetone and embedded in Durcupan ACM Araldite resin (Electron Microscopy Sciences, EMS, PA), using two pieces of ALCARE sheet (Ted Pella Inc.), which were situated and stabilized between two glass slides. The samples were polymerized for 48 h at 60°C.

### Serial Block-Face Scanning Electron Microscopy

Using a razor blade, the left and right ventricles were trimmed (Figure 1C) and mounted on a standard Gatan 3View aluminum specimen pin (EMS item # 75959-02) using silver conductive epoxy glue (Ted Pella Inc.) to electrically ground the tissue block. The sample was trimmed to a square or rectangular shape and positioned with a Leica ultramicrotome (Leica EM UC6, Buffalo Grove, IL, United States). The sample was then sputter-coated with a thin layer of 80/20 gold/palladium (Denton Vacuum DESK V sputter coater, NJ, United States). Coating was performed to further reduce any electrical charge during imaging. Serial block face (SBF) imaging was performed using a Gatan OnPoint BSE detector in a Zeiss GEMINI300 VP Field Emission Scanning Electron Microscope (FE-SEM) equipped with a Gatan 3View automatic microtome unit (Gatan, Pleasanton, CA, United States). The system was set to cut sections at 50 nm thickness. Imaging was performed utilizing Focus Charge Compensation nitrogen gas injection set at 40% (2.9E-03 mBar) to reduce imaging artifacts related to sample-charge. Images were collected after each round of sectioning from the block face using the SEM beam at 1.2 keV with a dwell time of 2.0 μs/pixel and a working distance of 7.4 mm. Each frame was 38 × 50 μm with a pixel size of 3.5 nm and slice thickness of 50 nm. Data acquisition occurred in an automated way using the Auto Slice and View G3 software to collect a 1 × 3 montage scan with a 15% (7.5 μm) overlap. A stack of 150 slices was aligned, stitched, and assembled using the Grid/Collection stitching plugin for ImageJ (Preibisch et al., 2009). A volume of 38 × 105 × 15 μm^3^ dimensions was obtained from the tissue block.

### Data Processing and Analysis

Data was acquired in an automated way using Gatan Digital Micrograph and analyzed with ORS Dragonfly software (Object Research Systems Inc.). For the experiments, 3 hearts per genotype were used, 3 control and 3 PKP2cKO hearts in total. Each heart was divided in the LV and RV and per ventricle we studied 2–3 regions of interest (ROI). Per ROI, 3–4 gap junction plaques were analyzed. Each gap junction plaque was analyzed throughout the entire structure (3D), meaning from the first plane until the last plane. Per plane (2D), we manually draw the area between Cx43 immunoreactive beads and length of the gap junction plaque, which was calculated by ORS Dragonfly software accordingly. The maximal width was measured in the widest area of the entire gap junction plaque. For 3D segmentation of the inner ID space, we traced the contour of the ID membrane. Hemiplaque length was manually drawn over the gap junction area that did not consist out of a consecutive “double” strand of Cx43 immunoreactive beads and calculated by ORS Dragonfly software.

## Statistics

Continuous variables are presented as box and whisker plots, reaching from the minimum to maximum value. Categorical variables are presented as percentage or total length. All data sets were tested for normal distribution by the Shapiro-Wilk and Kolmogorov-Smirnov tests, data were considered normally distributed when passing one of these two tests. Normally distributed data were compared using the Student’s *t*-test. Non-normally distributed data were compared using the Mann–Whitney Test. Statistical significance was assumed when *p* < 0.05. Statistical tests were performed using GraphPad Prism.

## Results

### FluoroNanogold Labeling of Connexin43 in Adult Murine Hearts

FluoroNanogold labeling in combination with silver enhancement was tested on thick vibratome (200 µm) sections of control adult murine hearts (Figure 1A). FluoroNanogold 546-labeled vibratome sections were imaged with light microscopy and Cx43 fluorescent labeling was clearly detectable in the perinexus (Figure 1B, left panel). Negative control stains, in which the primary antibody was omitted, were performed to confirm specificity of Cx43 fluorescent labeling; fluorescent signal appeared completely absent. Subsequently, these sections were processed for silver enhancement. Gold labeled Cx43 was distinctly visible in the gap junction plaques after silver enhancement. (Figure 1B, right panel). Pictures in both frames are from the same cardiac section. Further studies, focused on exploring alterations in the gap junction plaque morphology by loss of PKP2 expression, were carried out in left or right ventricular free walls separately (Figure 1C) using nanogold as marker.

### 3D Immuno-EM Reconstruction of Connexin43 Labeling

**FIGURE 2:**
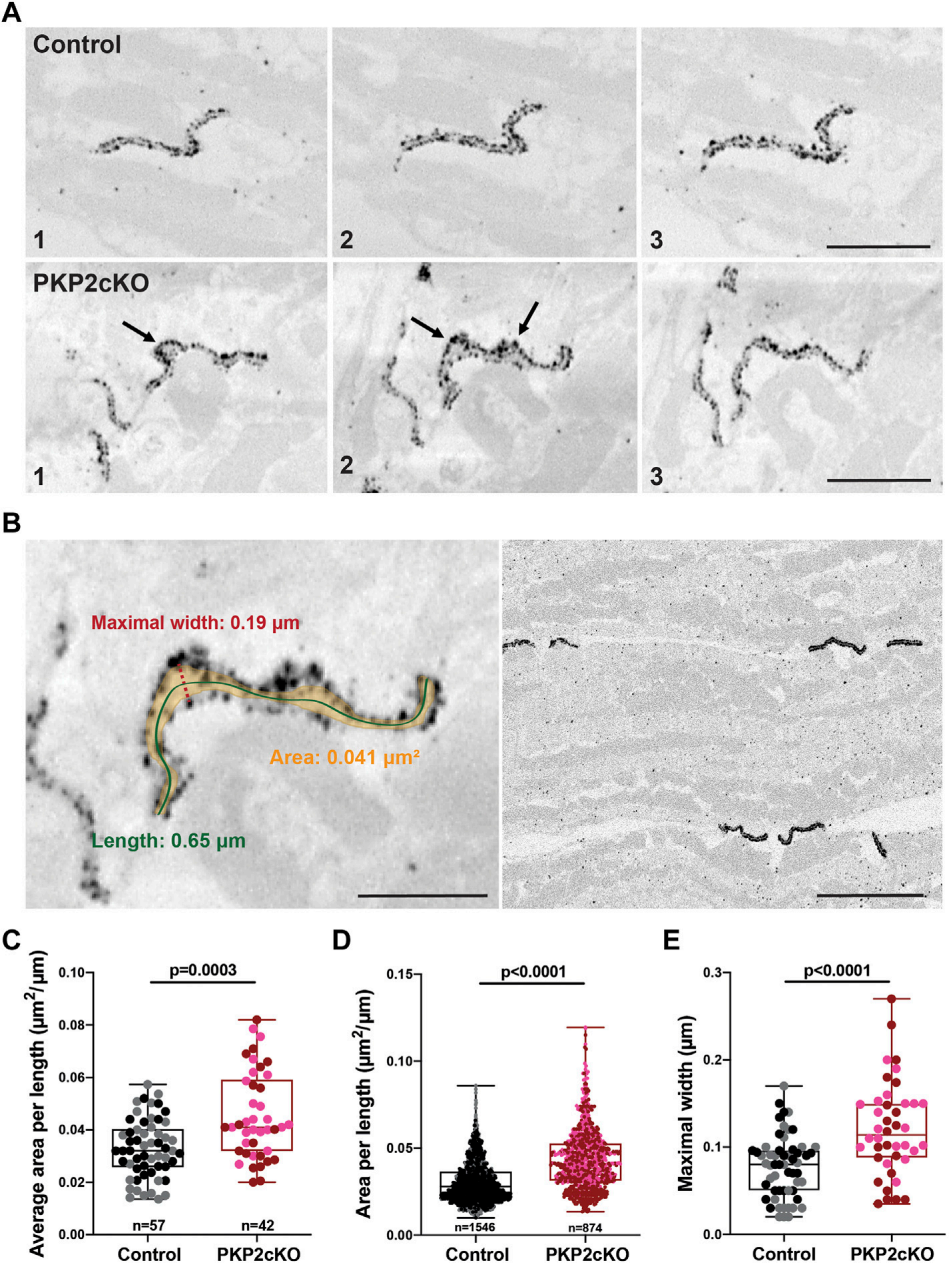
Gap junction plaque separation in adult hearts of Plakophilin-2 conditional knock out mice (PKP2cKO). **(A)** Representative examples of 3D Connenxin43 (Cx43) Immuno-Electron Microscopy images of a Control (top panel) and PKP2cKO (bottom panel) heart. The various frames (1–3) correspond to different section levels of the same sample. Arrowheads depict Cx43 hemichannels. Scale bar: 0.5 μm. **(B)** *Left panel*: Example of our method for quantifying parameters shown in **(C–E)**. Scale bar: 0.2 µm. *Right panel*: Low magnification example of multiple gap junction plaques in a Control heart sample. Scale bar: 0.5 µm. **(C,D)** Quantification of 2D surface between intercalated disc (ID) membranes in control and PKP2cKO hearts, corrected for ID length (Area per length), averaged per ID **(C)** and per individual section **(D)**. **(E)** Maximal width between ID membranes, per ID. **(C–E)**: Control; *n* = 57, PKP2cKO; *n* = 42. **(D)**: Control; *n* = 1,546, PKP2cKO; *n* = 874. Data presented as box and whisker (min.–max.) plot. Data points corresponding to left ventricular (LV) and right ventricular (RV) samples represented in separate colors; Control LV; black, Control RV; grey, PKP2cKO LV; red, PKP2cKO RV; pink. Significance in **(C,D)** as per Mann-Whitney test, in **(E)** per Student’s *t*-test.

**FIGURE 3:**
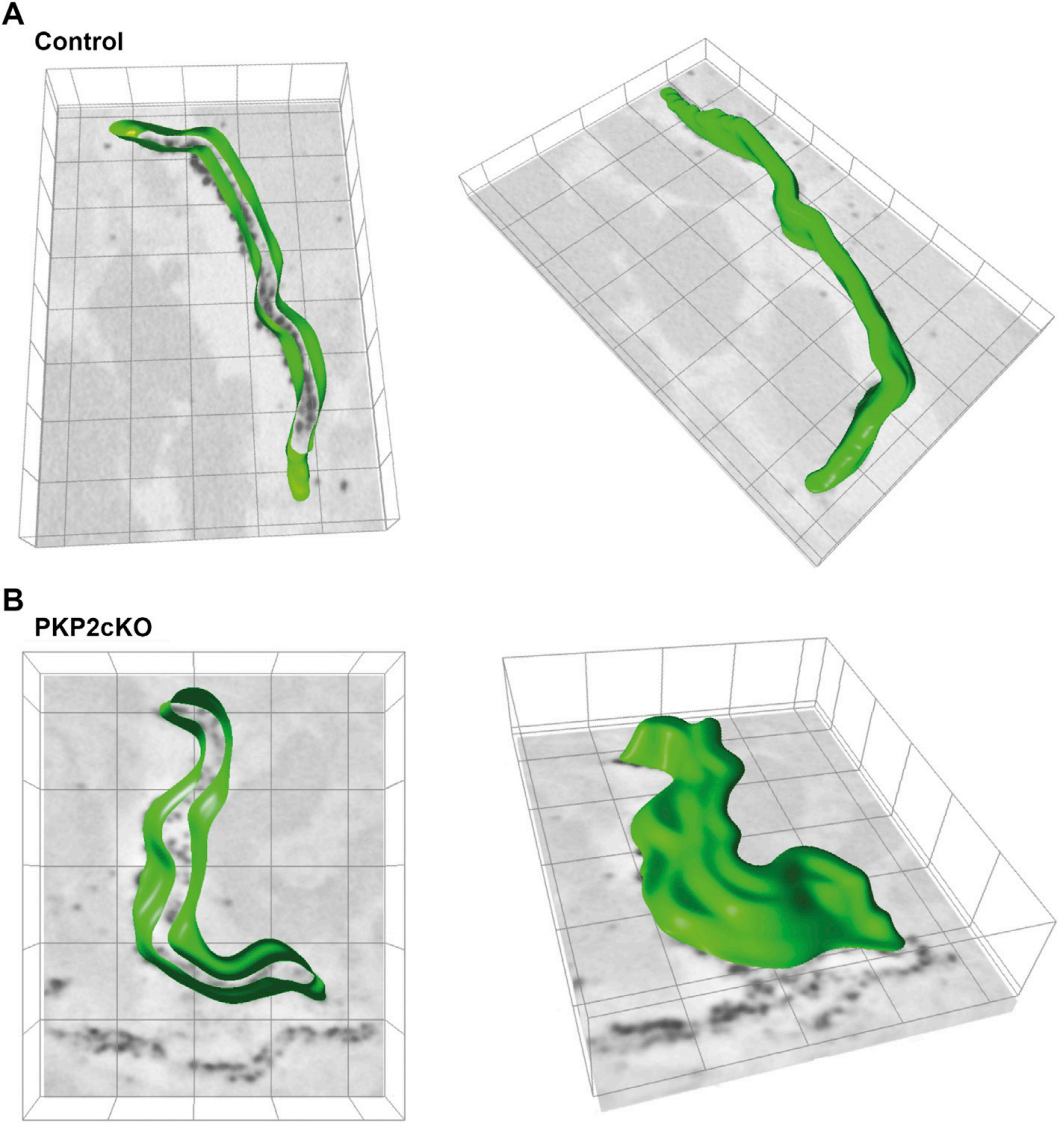
3D segmentation of the intercalated disc (ID) inner space in adult hearts of control and Plakophilin-2 conditional knock out mice (PKP2cKO). **(A)** *Left panel*: 3D segmentation, middle plane, of the inner space within the gap junction plaque of a control adult murine heart. The green line depicts Connexin43 immunoreactive beads segmented by ORS Dragonfly software. *Right panel*: Top view of the ID inner space 3D segmentation, from the same control sample presented on the left. **(B)** *Left panel*: 3D segmentation, middle plane, of the inner space within the gap junction plaque of a PKP2cKO adult murine heart. The green line depicts Connexin43 immunoreactive beads segmented by ORS Dragonfly software. *Right panel*: Top view of the ID inner space 3D segmentation, from the same PKP2cKO sample presented on the left. Please note widening of the intercellular space and areas where single Connexin43 immunoreactive beads lack opposing beads in close proximity in the PKP2cKO sample.

It is known that loss of *PKP2* expression impairs cell-cell adhesion and loss of cell adhesion destabilizes gap junctions (Bass-Zubek et al., 2008; Sato et al., 2011). In (Kim et al., 2019) we confirmed cell separation at the intercalated disc in hearts of PKP2cKO mice 14 dpi (Kim et al., 2019). In this study, we follow up on these observations and implement a quantitative approach. To characterize the perinexal morphology, we obtained 3D reconstructions of Cx43 in control and PKP2cKO murine hearts by nanogold labeling of ventricular free walls. These corresponding cardiac sections were imaged using SBF-SEM and images were collected with a Gatan 3View system. Reconstructed images allowed us to visualize the entire length of gap junction plaques, seen as two parallel, closely packed strings of Cx43-immunoreactive beads at the intercalated disc (Figure 2A upper panel, Figure 2B right panel, Figure 3A, Supplementary Video S1). Separation of these closely aligned Cx43 beads became apparent in the PKP2cKO 14dpi cardiac samples (Figure 2A bottom panel, Figure 3B, Supplementary Video S2), in both in the left and right ventricular free wall. Visualization of these morphological discrepancies was performed by 3D segmentation and conventional image processing (Figures 2, 3).

### Visualization of Orphan Connexin43 Hemichannels in PKP2cKO Hearts

In Figure 2A, the various frames correspond to different section levels of the same samples, either control, or from a PKP2cKO heart. These pictures emphasize morphological alterations throughout the gap junction plaque structure and highlight the importance of 3D reconstructions for quantitative analyses. Widening of the intercellular space and areas where single Cx43 immunoreactive beads lack opposing beads in close proximity, could be clearly observed in PKP2cKO hearts (Figure 2A bottom panel). These observations indicate the presence of orphan Cx43 hemichannels in adult PKP2cKO murine hearts, as suggested in (Kim et al., 2019). To measure the extent of perinexal remodeling, we performed a quantitative analysis on samples from control and PKP2cKO mice; multiple gap junction plaques per heart were included.

### Quantification of Perinexal Alterations in PKP2cKO Hearts

**FIGURE 4:**
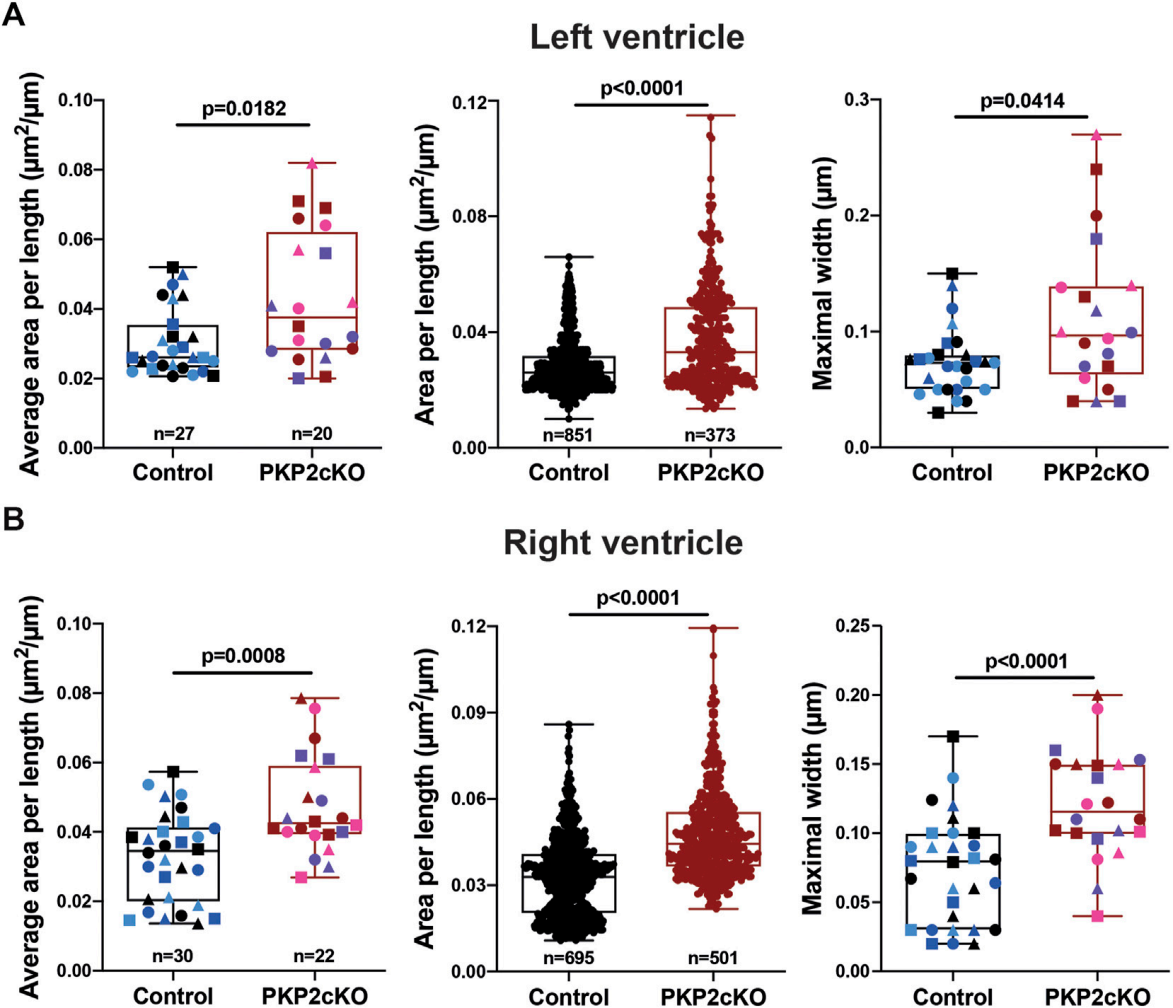
Gap junction plaque separation in left and right ventricular samples of Plakophilin2 conditional knock out hearts (PKP2cKO). **(A)** Quantification of 2D surface between intercalated disc (ID) membranes in left ventricular samples of control and PKP2cKO hearts, corrected for ID length (Area per length), averaged per ID (*left panel*) and per individual section (*middle panel*). *Right panel*: Maximal width between ID membranes, per ID. *Left and right panel*: Control; *n* = 27, PKP2cKO; *n* = 20. Middle panel: Control; *n* = 851, PKP2cKO; *n* = 373. Data presented as box and whisker (min.– max.) plot. Colors depict data points corresponding to different animals. Shapes depict data points corresponding to differenct regions of interest. Significance as per Mann-Whitney test. **(B)** Quantification of 2D surface between intercalated disc (ID) membranes in right ventricular samples of both groups, corrected for ID length (Area per length), averaged per ID (*left panel*) and per individual section (*middle panel*). *Right panel*: Maximal width between ID membranes, per ID. *Left and right panel*: Control; *n* = 30, PKP2cKO; *n* = 22. *Middle panel*: Control; *n* = 695, PKP2cKO; *n* = 501. Data presented as box and whisker (min.–max.) plot. Colors depict data points corresponding to different animals. Shapes depict data points corresponding to different regions of interest. Significance in left and middle panel as per Mann-Whitney test, right panel as per Student’s *t*-test.

Gap junction plaques in 3D were analyzed using ORS Dragonfly software. The inner space within the gap junction plaque was segmented, allowing us to quantify the surface area per plane and throughout the entire structure (Figure 2B left panel, Figure 3). As gap junction plaques differed largely in depth and length, we decided to measure in addition the gap junction plaque length and maximal width between the ID membranes (Figure 2B left panel). We corrected the surface area of the total gap junction plaque (average area), or individual frame (area), for the gap junction plaque length (average area per length). Very significant differences in the average area per length, area per length of individual frames and maximal width between ID membranes were observed between control and PKP2cKO hearts (Figures 2C–E). In Figures 2C–E, pooled data of the left ventricle (LV) and RV are presented, in Figure 4 separate analysis of the LV and RV is shown. When quantifying these parameters in both ventricular walls separately, the differences between control and PKP2cKO hearts appeared to be very significant as well (Figures 4A,B).

### Detection of Connexin43 Hemiplaques in PKP2cKO Hearts

**FIGURE 5:**
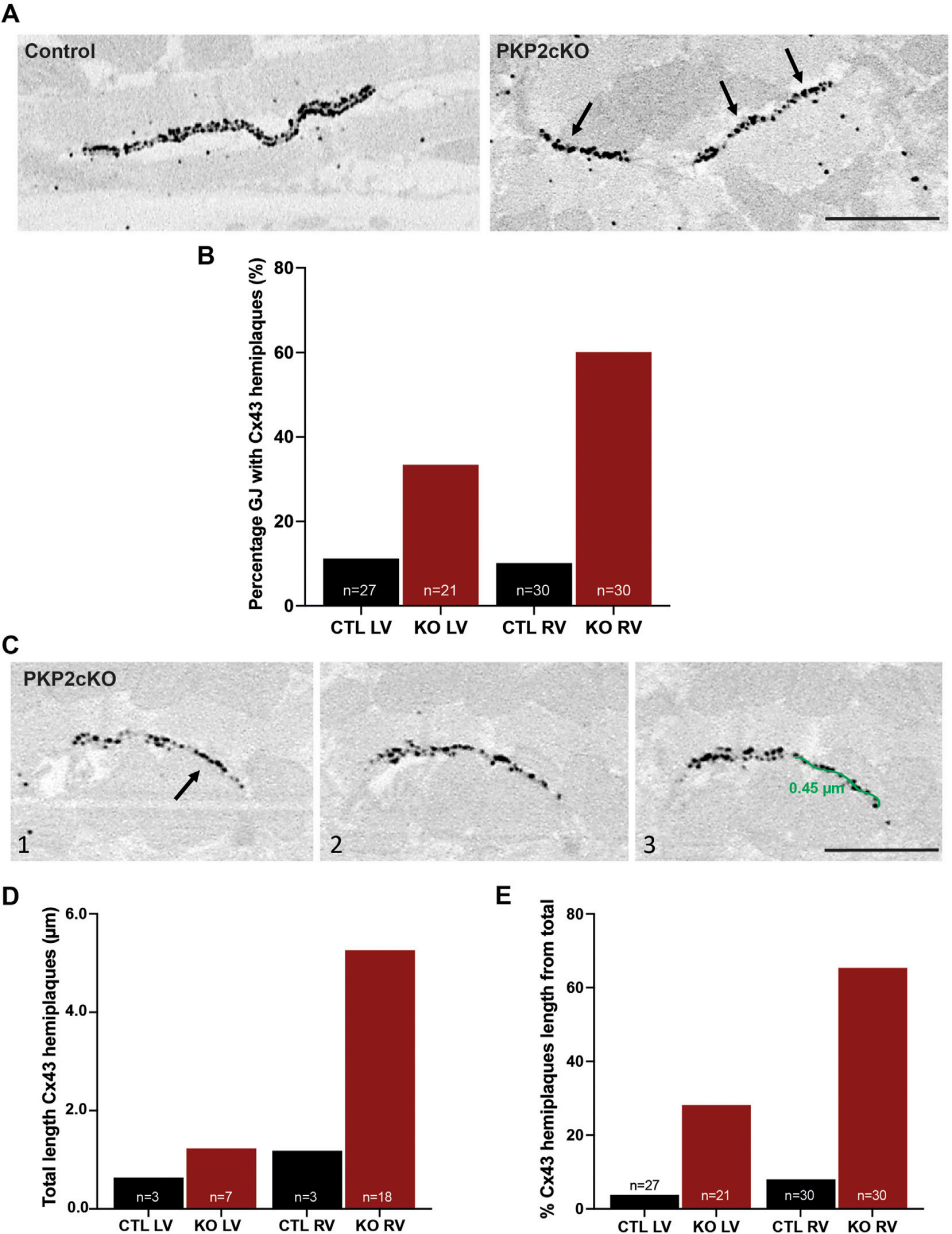
Connexin43 hemiplaques in adult hearts of Plakophilin-2 conditional knock out mice (PKP2cKO). **(A)** Representative examples of gap junction plaques in control and PKP2cKO hearts, emphasizing in the right panel Connexin43 (Cx43) hemiplaques in a PKP2cKO ventricular sample. Arrowheads point out Cx43 hemiplaques. Scale bar: 0.5 µm. **(B)** Quantification of the percentage of gap junction plaques containing Cx43 hemiplaques in control and PKP2cKO hearts, left ventricular (LV) and right ventricular (RV) samples separately. Data presented as percentage of total pool. Control LV; *n* = 27, PKP2cKO LV; *n* = 21, Control RV; *n* = 30, PKP2cKO RV; *n* = 30. **(C)** Representative examples of 3D Cx43 Immuno-Electron Microscopy images of a PKP2cKO heart presenting a Cx43 hemiplaque. The various frames (1–3) correspond to different section levels of the same samples. Arrowheads point out Cx43 hemiplaques. Scale bar: 0.5 µm. **(D)** Quantification of the total length of Connexin43 hemiplaques in control and PKP2cKO hearts, LV and RV samples separately. Data presented as total length of total pool. Control LV; *n* = 3, PKP2cKO LV; *n* = 7, Control RV; *n* = 3, PKP2cKO RV; *n* = 18. **(E)** Quantification length of Connexin43 hemiplaques in control and PKP2cKO hearts, corrected for the total length of the gap junction plaque. LV and RV samples are quantified separately. Data presented as percentage of total length per group. Control LV; *n* = 27, PKP2cKO LV; *n* = 21, Control RV; *n* = 30, PKP2cKO RV; *n* = 30.

In addition to bulging of the intercellular space and disconnect of opposing Cx43 channels, in PKP2cKO hearts we also observed gap junction plaques that, for some length, showed only one single strand of Cx43 immunoreactive beads (Cx43 hemiplaques; see Figure 5A). We quantified the percentage of gap junction plaques in which Cx43 hemiplaques were observed. As shown in Figure 5B, Cx43 hemiplaques were present in both ventricular walls of PKP2cKO hearts, but to a higher extent in the RV (Figure 5B). In addition, we quantified the total length of the Cx43 hemiplaque as a function of genotype and for both RV and LV (Figures 5C,D). The data show that the total length of Cx43 hemiplaques in the RV was over 3 times longer in PKP2cKO than in control hearts, or in the LV of either control or PKP2cKO hearts. When corrected by the total length of the gap junction plaque, hemiplaques occupied a higher proportion of the areas of Cx43 immunoreactivity in PKP2cKO-derived tissue than in controls, with the RV wall of PKP2cKO hearts showing the highest values.

## Discussion

Over the past years, several investigators have studied loss of desmosomal integrity in hearts of ARVC patients by desmosomal protein mutations (Basso, 2006; Gomes et al., 2012; Noorman et al., 2013). Functional data has been largely supported by immunohistochemistry labeling of desmosomal proteins, though the complete structure of the PKP2-deficient ID in three dimensions remains unresolved. As a first step, here we describe the complete spatial ultrastructure of the gap junction plaques in control, and in PKP2-deficient myocytes prior to the development of a structural disease.

Studies in cell culture systems and in tissue from ARVC-affected patients have previously indicated that loss of expression or mutations in PKP2 can alter the structure and function of gap junctions (Oxford et al., 2007; Saffitz, 2009; Asimaki and Saffitz, 2014). Previous studies showed that PKP2 deficiency in the murine heart increases Cx43-mediated membrane permeability, and that the latter may facilitate ATP efflux (Cerrone et al., 2018) and excessive entry of Ca^2+^ into the cells (Kim et al., 2019). It is also known that Cx43 hemichannels reside in the perimeter of the gap junctions (Rhett et al., 2011) and are capable to flicker with very low probability when undocked (Contreras et al., 2003; Verma et al., 2009). Consistent with those observations, ablation of Cx43 prevented the accumulation of Ca^2+^_i_, and GAP19, a peptide known to block Cx43-Hs without affecting junctional conductance, normalized the intracellular Ca^2+^ homeostasis (Kim et al., 2019). These data support the hypothesis that Cx43-Hs are likely important players in the arrhythmogenic/cardiomyopathic phenotype of these mice. Here, we provide structural data in support of the notion that Cx43 hemichannels are present with a higher abundance in the membrane of PKP2-deficient hearts. We thus postulate that these single strings of Cx43 immunoreactive molecules are the structural counterpart to the excess Cx43 hemichannels detected by functional assays. Of note, previous studies have shown an absence of changes in Cx43 abundance (Cerrone et al., 2017; Kim et al., 2019). Given the timing of our experiments vis a vis the development of a PKP2-dependent cardiac phenotype in these mice, we conclude that the nanostructural changes in the ID described here are among the earliest events following loss of PKP2 expression.

We show that the multimodal imaging application offered by nanogold or FluoroNanogold labeling can be applied for vEM in thick cardiac sections. Highlighting it as novel tool for future use in 3D imaging of protein localization in different tissue types. FluoroNanogold is a probe containing two different markers (Takizawa and Robinson, 2000). As such, it opens the possibility of imaging the same sample at both the optical and the EM level (Robinson and Vandré, 1997; Takizawa and Robinson, 2000). The method can be used for multimodal studies, either on different cell types or on the same cell, visualized by correlative microscopy. The very small size of the gold atom clusters allows efficient absorption within fixed tissues prior to embedding (Robinson and Vandré, 1997). Quantum dots are largely used to identify and localize proteins simultaneously with different imaging approaches; however, they can only penetrate up to 4 μm deep into the tissue (Giepmans et al., 2005). Our experiments show that nanogold could easily penetrate in up to 20 μm on both sides of the cardiac vibratome sections. The use of nanogold and FluoroNanogold labeling in adult heart therefore represents a step forward in the development of novel methodologies for visualization of specific protein aggregates in native tissue, at nanometric resolution and in three dimensions.

Making use of FIB-SEM, we previously identified a nanometric separation of the ID membrane in RV samples of PKP2cKO mice (Kim et al., 2019). In hearts of heterozygous PKP2 knock out mice, we implemented high-pressure freezing tissue preservation for tomographic electron microscopy (tomographic EM) purposes (Leo-Macías et al., 2015). Besides clear detection of ID membrane separation, ultrastructural characteristics of the intercellular space could be visualized as well. Tomographic EM, however, did not allow detailed identification of individual gap junction plaques. Labeling Cx43, prior to imaging, is therefore critical to detect orphan hemichannels and asymmetric gap junctions in the ID membrane. In this study, IEM allowed us to examine both parameters (separation of the ID membrane and presence of Cx43-Hs) in parallel, adding an extra visual component to the previously obtained microscopy and functional data reported in Kim et al.

We emphasize that our experimental model (PKP2cKO) neither is intended to “recapitulate” ARVC nor is intended to be a surrogate for studies of human hearts affected with disease. This is in fact true for all animal or cellular models of ARVC that have been published, and an obvious consequence of the limitations inherent to the study of a human disease. The significance of our studies is not in having reproduced conditions that lead to human ARVC, but in having studied a molecule that, when mutated, causes the disease. Whether the structural changes described here occur in human hearts with PKP2 mutations remains undefined. Extrapolation from our data to the specific case of ARVC needs to be done with caution. That said, our model has shown consistency with human informatics data (Montnach et al., 2018) and has been useful in proposing therapeutic approaches to patients with ARVC (e.g., the pilot clinical trial on use of flecainide in ARVC patients; NCT03685149).

In conclusion, the results described in this study support the notion that, at this stage in the progress of the phenotype (i.e., before histologically-detectable disease), we detect a nanometric separation of the gap junction plaque, and the presence of unopposed Cx43-immunoreactive single strings (orphan Cx43-Hs). We propose that these orphan hemichannels are the structural correlate to the functional Cx43-Hs previously detected by (Kim et al., 2019). Our data illustrate the value and the potential of the novel methodology described here for visualization. It also provides further support to the notion that the gain-of-function of Cx43-Hs constitutes an important arrhythmogenic substrate in the setting of PKP2 deficiency. We speculate that block of Cx43-Hs may represent a novel and effective therapeutic strategy to prevent life-threatening arrhythmias in patients with PKP2 deficiency, particularly in the concealed stage of the disease.

## Data Availability

The raw data supporting the conclusion of this article will be made available by the authors, without undue reservation.

## Abbreviations

ARVC, arrhythmogenic right ventricular cardiomyopathy; BSA, bovine serum albumin; Ca^2+^, calcium; Cx43, connexin43; Cx43-Hs, connexin43 hemichannels; dpi, days post tamoxifen injection; EM, electron microscopy; FE-SEM, field emission-scanning electron microscope; IEM, immuno-electron microscopy; LV, left ventricle; PBS, phosphate buffer saline; PFA, paraformaldehyde; PKP2, plakophilin-2; ID, intercalated disc; PKP2cKO, cardiomyocyte-specific, tamoxifen-activated, PKP2 knockout; RV, right ventricle; SBF-SEM, serial block-face scanning electron microscopy; SEM, scanning electron microscopy; TAM, tamoxifen; TEM, transmission electron microscopy; vEM, volume electron microscopy.
